# Bioactive Components of *Parthenocissus quinquefolia* with Antioxidant and Anti-Inflammatory Properties: A Systematic Review

**DOI:** 10.3390/antiox15020169

**Published:** 2026-01-27

**Authors:** Álvaro Becerra, Felipe Soto, Alejandro Vallejos, Daniela Millán, Juan José Valenzuela-Fuenzalida, Jose E. Leon-Rojas, Manuel E. Cortés

**Affiliations:** 1 Escuela de Fonoaudiología & Departamento de Ciencias Químicas y Biológicas, Facultad de Ciencias de la Salud, Universidad Bernardo O’Higgins, Santiago 8370854, Chile; alvaro.becerra@ubo.cl (Á.B.); f.soto.guerrero@gmail.com (F.S.); vallejosa@docente.ubo.cl (A.V.); 2 Escuela de Nutrición y Dietética & Centro Integrativo de Biología y Química Aplicada (CIBQA), Facultad de Salud, Universidad Bernardo O’Higgins, Santiago 8370854, Chile; daniela.millan@ubo.cl; 3 Departamento de Morfología, Facultad de Medicina, Universidad Andrés Bello, Santiago 8370146, Chile; juan.kine.2015@gmail.com; 4 Grupo de Investigación Bienestar, Salud y Sociedad, Escuela de Psicologia y Educación, Universidad de Las Américas, Quito 170124, Ecuador; 5 Dirección de Investigación, Vicerrectoría Académica, Universidad Bernardo O’Higgins, Santiago 8370854, Chile; manuel.cortes@ubo.cl

**Keywords:** Nrf2/ARE pathway, NF-κB signaling, secondary metabolites, phenolic profile, phytochemical analysis, bioactive compounds, Vitaceae family

## Abstract

**Background:** *Parthenocissus quinquefolia* (Virginia creeper), widely distributed and used in Chile, lacks a systematic characterization of its bioactive components. This study synthesizes the evidence on the phytochemical composition and biological activities of *P. quinquefolia*, with emphasis on metabolites involved in redox regulation and inflammation. **Methods:** A systematic review was conducted following PRISMA 2020 guidelines. Searches were performed across four electronic databases, including original studies reporting antioxidant and anti-inflammatory effects. **Results:** Of 665 records identified, 14 studies met the inclusion criteria. Phytochemical analyses revealed phenolic compounds, particularly flavonoids (e.g., catechin, epicatechin, gallocatechin, epigallocatechin, quercetin, rutin, isoquercitrin, myricetin, luteolin, naringin) and stilbenes (e.g., ε-viniferin, miyabenol C). These metabolites exhibit antioxidant activity through ROS scavenging, metal chelation, and Nrf2/ARE activation. Anti-inflammatory effects were attributed to the downregulation of NF-κB, AP-1, and MAPK signaling, inhibition of NLRP3 inflammasome activation, and suppression of COX-2/iNOS expression. Conclusions: *P. quinquefolia* is a rich source of phenolic metabolites with robust antioxidant and anti-inflammatory mechanisms. The consistency of molecular responses across studies highlights its potential as a promising candidate for phytotherapeutic development targeting oxidative stress and inflammatory pathways.

## 1. Introduction

*Parthenocissus quinquefolia* (L.) Planch., commonly known as *Virginia creeper*, is a member of the Vitaceae family and is indigenous to North America; however, it is now cultivated and distributed worldwide, including in Chile through horticultural nurseries and ornamental landscaping projects [1,2]. Although P. quinquefolia is exotic to the Southern Cone, recent interest in introduced ornamental and medicinal plants in Chile alongside ongoing research into native species with antioxidant potential (e.g., *Aristotelia chilensis*, *Berberis microphylla*, *Peumus boldus*) [3,4] suggests that *P. quinquefolia* may represent a complementary phytochemical resource. Its local availability further enhances its potential for phytochemical research and future biotechnological applications. Historically, *P. quinquefolia* has been used by Native American populations for a wide range of medicinal purposes, including treatments for inflammatory conditions, metabolic disorders, and infectious processes [5]. More recently, experimental studies have reported antioxidant, anti-inflammatory, metabolic, antimicrobial, and anticancer properties associated with extracts obtained from different plant organs [6,7,8,9,10,11]. Collectively, these findings suggest potential protective effects against cancer progression, as well as antibacterial, antiviral, antidiabetic, antirheumatic, hypocholesterolemic, gastrointestinal, and diuretic activities. Collectively, these findings suggest potential protective effects against cancer progression, as well as antibacterial, antiviral, antidiabetic, antirheumatic, hypocholesterolemic, gastrointestinal, and diuretic activities. These biological properties are largely attributed to the presence of secondary metabolites bioactive compounds synthesized by plants in response to biotic and abiotic stimuli, enabling adaptation to environmental stressors [12]. These compounds are widely utilized in the pharmaceutical, cosmetic, nutraceutical, food, and agrochemical industries [13,14,15]. Consequently, the ethnobotanical and pharmacological characterization of introduced and native species has become increasingly relevant for identifying compounds with biomedical applications.

Given that many of these biological activities are mechanistically linked to redox imbalance and immune modulation, it is pertinent to consider the role of inflammation in this context. Inflammation represents a complex biological response to cellular and tissue injury caused by pathogens (e.g., viruses, bacteria, fungi), chemical irritants, physical agents, or dysregulated immune activity. When unresolved, inflammation may become chronic, promoting oxidative stress, cellular damage, and molecular remodeling associated with carcinogenesis, accelerated aging, metabolic disease, and increased mortality [16,17]. Considerable evidence has demonstrated that secondary metabolites exert antioxidant and anti-inflammatory effects through redox modulation, reactive oxygen species (ROS) scavenging, mitochondrial protection, and regulation of pro-inflammatory signaling pathways such as NF-κB, COX-2, and iNOS [18,19,20,21]. The abundance of phenolic compounds with antioxidant and anti-inflammatory properties reported in *P. quinquefolia* suggests that this species may contribute to the identification of novel therapeutic targets. Therefore, the objective of this systematic review is to summarize the available evidence regarding the composition, distribution, and biological effects of secondary metabolites with anti-inflammatory potential isolated from or identified in *P. quinquefolia*.

The abundance of compounds with antioxidant and anti-inflammatory properties in *P. quinquefolia* suggest its potential for the identification of novel therapeutic targets. The objective of this review is to provide an overview of the reported composition, distribution, and biological effects of secondary metabolites with anti-inflammatory properties in *P. quinquefolia.*

**Figure 1:**
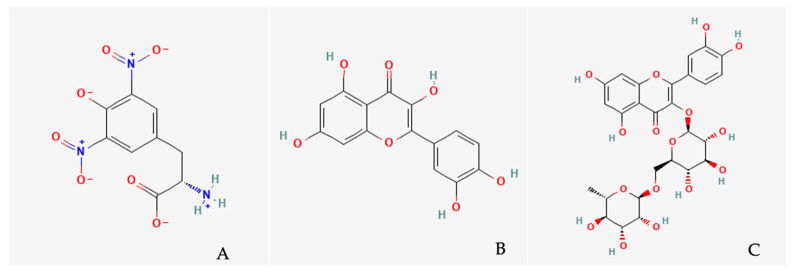
Chemical structures of key phenolic metabolites identified in *Parthenocissus quinquefolia*. (**A**) Quercetin, (**B**) Rutin, (**C**) ε-Viniferin.

Representative structures of phenolic metabolites identified in *P. quinquefolia* are shown in Figure 1 to illustrate the chemical diversity of flavonoids and stilbenoids present in the species.

## 2. Materials and Methods

### 2.1. Method Designs

This study was guided by the checklist of proposed elements according to Preferred Reporting Items for Systematic Reviews and Meta-Analyses (PRISMA) 2020 [22]. The registration DOI in Open Science Framework (OSF) is https://osf.io/hx8nw (Accessed 4 January 2026).

### 2.2. Selection Criteria

Studies eligible for inclusion are original research articles that provide information on the anti-inflammatory or antioxidant effects, as well as other beneficial health effects, of the plant species *P. quinquefolia*, or any of its other names mentioned in the search terms (*Ampelopsis quinquefolia*, *Hedera quinquefolia*, *Vitis quinquefolia*, *Virginia creeper*), published in English.

### 2.3. Information Source and Search Strategies

A systematic search was conducted in PubMed, Scopus, Web of Science, and Google Scholar from inception to May 2025. Additional records were identified through reference screening. No publication date restrictions were applied. Language was restricted to English and only original research articles were eligible. The following Boolean search strategy was used: (“*Parthenocissus quinquefolia*” OR “*Ampelopsis quinquefolia*” OR “*Hedera quinquefolia*” OR “*Vitis quinquefolia*” OR “*Virginia creeper*”) AND (“antioxidant” OR “anti-inflammatory” OR “bioactive” OR “phytochemical” OR “stilbene” OR “phenolic” OR “flavonoid”). Database-specific search strings are provided in Supplementary Material (Table S1), in-cluding search dates, Boolean operators, and applied filters.

### 2.4. Study Selection

All records identified through database searching were imported into a reference manager and duplicates were removed. The screening process followed the PRISMA 2020 recommendations and was conducted in two stages. In the first stage, two reviewers independently screened titles and abstracts. Records were excluded at this stage if they met any of the following criteria: (1) non-original publications (e.g., reviews, conference abstracts, book chapters), (2) studies not focusing on *Parthenocissus quinquefolia*, or (3) absence of biological, phytochemical, antioxidant, or anti-inflammatory outcomes. Full-text articles were then retrieved for all records that passed the initial screening. In the second stage, the same reviewers independently assessed full-text eligibility. Full-text exclusions were applied for the following reasons: (1) full text not accessible, (2) language not meeting inclusion criteria, (3) absence of relevant biological outcomes, or (4) unclear plant identification. Discrepancies at any screening stage were resolved by discussion and consensus. No automation tools were used during the selection process.

### 2.5. Methodological Quality Assessment

Because the included evidence consisted of phytochemical characterizations and non-clinical experimental studies (in vitro/in vivo), traditional clinical risk of bias tools (e.g., RoB 2, ROBINS-I) were not applicable. Instead, methodological quality was assessed using criteria adapted for non-clinical research, including: (1) botanical identification, (2) extraction reporting, (3) analytical validation, (4) use of controls in bioassays, and (5) replication reporting. Two reviewers independently assessed methodological quality, and disagreements were resolved by consensus.

## 3. Results

**Figure 2:**
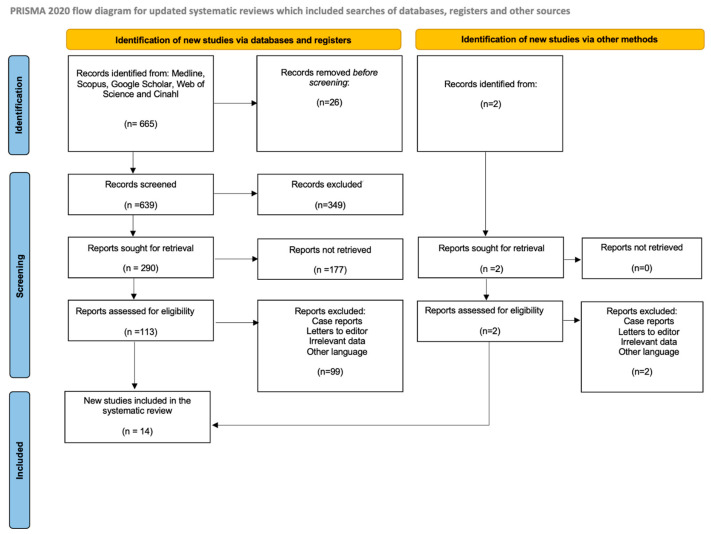
PRISMA 2020 flow diagram illustrating the selection of studies included in the review. Adapted from Page et al. (2021) [22].

A total of 665 records were identified through database searching, and an additional 2 records were identified through other sources. Before screening, 26 records were removed. A total of 639 records were screened at the title and abstract level, resulting in the exclusion of 349 records for not meeting the inclusion criteria. Full texts were sought for 290 records, of which 177 could not be retrieved. The remaining 113 full-text reports were assessed for eligibility. Of these, 99 were excluded due to being case reports, letters to the editor, presenting irrelevant data, or being published in a non-eligible language. In parallel, two additional reports identified through other sources were assessed, both of which were excluded for the same reasons. Ultimately, 14 studies met the inclusion criteria and were incorporated into the systematic review. The updated PRISMA 2020 flow diagram is shown in Figure 2. All included studies were written in English and consisted exclusively of laboratory work without human or animal intervention. The studies originated from a broad geographical distribution, including Pakistan (n = 2), Ukraine (n = 2), and one study each from Japan, China, Thailand, Tunisia, India, Iran, Turkey, the United States, Hungary, and Saudi Arabia. The included research focused on phytochemical analyses and non-clinical assays performed on different plant parts of *P. quinquefolia*, including leaves, fruits, stems, and shoots. Analytical approaches varied, encompassing colorimetric assays, chromatographic profiling (e.g., HPLC, HPLC-MS), and spectrophotometric quantification. Experimental models included antioxidant capacity assays (e.g., DPPH, ABTS, FRAP) and other non-interventional in vitro assays. The main characteristics of the included studies are summarized in Table 1.

The included studies focused on extracting and determining the chemical composition of different parts of *P. quinquefolia*, as well as their possible biological effects. Based on the extracted information, the properties of the species were categorized according to: (i) the plant parts used (stems, leaves, fruits, shoots), (ii) the type of extracts obtained, and (iii) the biological effects evaluated by the authors. These categories are summarized in Table 2.

## 4. Discussion

### 4.1. Phytochemical Profile of Parthenocissus quinquefolia

Available evidence indicates that *P. quinquefolia* contains a diverse spectrum of phenolic secondary metabolites, particularly flavonoids and stilbenoids, which represent the most consistently reported bioactive constituents of the species (Table 2). Phenolic compounds are biosynthesized through the phenylpropanoid pathway and are characterized by at least one aromatic ring bearing hydroxyl substituents. Structurally, they encompass several subclasses, of which flavonoids and stilbenes are the most relevant in *P. quinquefolia* due to their antioxidant and anti-inflammatory potential [32,33,34,35,36,37,38].

#### 4.1.1. Flavonoids

Flavonoids are low-molecular-weight polyphenols defined by a C6–C3–C6 backbone. In *P. quinquefolia*, flavonoids reported include flavonols (e.g., quercetin and glycosylated derivatives) and flavan-3-ols (e.g., catechin and epicatechin), mostly detected in leaves, shoots and fruits. These compounds have been associated with radical-scavenging activity, metal chelation, and modulation of redox-sensitive signaling pathways such as NF-κB and Nrf2/ARE in broader pharmacological literature, providing mechanistic plausibility for the antioxidant and anti-inflammatory effects observed in the plant.

Among detected flavonols, quercetin and rutin are particularly relevant. Quercetin has been widely characterized as an antioxidant through direct ROS scavenging as well as activation of endogenous enzymatic defenses via Nrf2, whereas glycosylated forms such as rutin display enhanced stability and documented anti-inflammatory effects via NF-κB inhibition [39,40,41,42,43,44,45,46,47,48,49,50,51,52,53,54,55,56,57]. Although these mechanistic properties have been largely demonstrated in non-*Parthenocissus* systems, their presence in *P. quinquefolia* suggests functional relevance within its phytochemical matrix.

Flavan-3-ols such as catechin and epicatechin have also been reported. These compounds possess catechol-type moieties that enhance redox activity and exhibit documented interactions with endothelial nitric oxide synthase and pro-inflammatory cytokine signaling, supporting a role in vascular redox modulation [58,59,60,61,62,63,64,65,66,67,68,69,70]. Their occurrence in *P. quinquefolia* aligns with the polyphenolic profiles documented in other Vitaceae.

#### 4.1.2. Stilbenoids

Stilbenes constitute another major phenolic class in *P. quinquefolia*. The species contains resveratrol and a range of resveratrol-derived oligomers (e.g., parthenocissins and ε-viniferin), expanding its stilbenoid repertoire beyond that of many related taxa [24,71,72,73,74,75,76,77,78,79,80,81,82,83,84,85,86,87,88,89,90,91,92]. Structural oligomerization enhances π-electron delocalization and increases the number of accessible phenolic hydroxyl groups, properties associated with superior radical-scavenging and anti-inflammatory capacity relative to monomeric resveratrol [93,94,95,96,97,98,99,100,101,102,103,104,105].

Notably, parthenocissin-type oligomers have been isolated from the genus *Parthenocissus*, including dimers and higher-order oligomers. Although their functional characterization remains limited in *P. quinquefolia*, studies from other Vitaceae demonstrate inhibition of NF-κB and COX-2/iNOS expression by related oligomers such as ε-viniferin, supporting mechanistic plausibility for anti-inflammatory effects [106,107,108,109,110,111,112,113,114,115,116,117,118].

**Figure 3:**
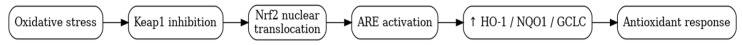
Proposed mechanism for antioxidant activation through the Nrf2/ARE pathway by phenolic metabolites identified in *Parthenocissus quinquefolia*. Phenolic compounds such as quercetin and myricetin promote Nrf2 nuclear translocation and induction of antioxidant enzymes (e.g., HO-1, SOD, CAT), contributing to redox homeostasis.

**Figure 4:**
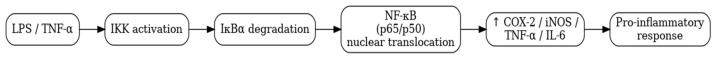
Proposed mechanism for anti-inflammatory modulation through NF-κB inhibition by phenolic metabolites identified in *Parthenocissus quinquefolia*. Compounds including catechins, rutin and ε-viniferin reduce IκBα degradation and NF-κB nuclear translocation, downregulating COX-2, iNOS and pro-inflammatory cytokines.

Among the studies included, antioxidant activity was evaluated exclusively through chemical in vitro assays, mainly DPPH, ABTS and FRAP, demonstrating relevant radical-scavenging and reducing capacity of extracts from leaves, shoots and fruits. However, **no** studies were found assessing antioxidant effects in cell-based models, animal models or clinical settings, and no biomarker-based evaluations (e.g., lipid peroxidation, protein oxidation, antioxidant enzymes) have been reported to date for *P. quinquefolia*. This gap prevents extrapolation of the in vitro antioxidant findings to physiological or therapeutic contexts and highlights the need for validation in biological systems. The findings of this review support that *P. quinquefolia* represents a relevant source of phenolic metabolites, in particular flavonoids and stilbenoids, capable of modulating central redox-inflammatory pathways such as NF-κB, COX-2/iNOS and the generation of PGE_2_/NO. This body of evidence provides biological plausibility to the antioxidant and anti-inflammatory effects attributed to the species. The most direct phytochemical and functional information derive from the studies on leaves, shoots and fruits, where flavones, flavonols and catechins (i.e., quercetin, quercetin-3-β-glucoside, rutin, catechin and epicatechin) have been identified and quantified, together with antioxidant activity evaluated by chemical assays. Taken together, this background confirms that the species harbors a complex polyphenolic matrix with significant bioactive potential (Figure 3 and Figure 4) [24,31].

A noteworthy aspect is the identification of resveratrol in *P. quinquefolia* [119], together with novel resveratrol oligomers, such as parthenocissin M and N [18]. These findings expand the stilbenoid repertoire of the species and suggest that part of its biological activity could be attributed to these higher-order assemblages. These oligomers exhibit distinct biological profiles compared to the monomer [24,120]. From a mechanistic perspective, the Vitaceae literature provides elements that enable the formulation of reasonable hypotheses without undue extrapolation. For example, in *Vitis heyneana* the *trans-ε-viniferin* dimer has been shown to suppress NF-κB activation and reduce COX-2/iNOS expression, thereby decreasing the dose-dependent production of PGE_2_ and NO in LPS-stimulated RAW 264.7 macrophages [118]. Given that *P. quinquefolia* contains stilbenoids and shares biosynthetic pathways with *Vitis*, including the production of viniferins and miyabenols [24,120,121], it is plausible that analogous oligomers present in this species contribute to anti-inflammatory effects through convergent molecular targets. In this context, the trimer miyabenol C, isolated and characterized in different Vitaceae [24,121,122], has been associated with neuroprotective effects involving β-secretase inhibition and β-amyloid reduction, indicating a functional spectrum that goes beyond redox modulation and reaches key proteases in amyloidogenesis [123]. Although these results derive from studies on *Vitis thunbergii var. taiwaniana* [123], they reinforce the idea that resveratrol oligomers may exhibit bioactivities superior or complementary to those of the monomer and may contribute to part of the effects reported in *P. quinquefolia*. With respect to flavonoids, the compounds detected in *P. quinquefolia*, such as rutin, quercetin and its glycosides (e.g., quercetin-3-O-alpha-L-rhamnoside) [119], catechin, epicatechin, and luteolin in the fruits, are supported by substantial mechanistic evidence. Quercetin and its glycosylated derivatives modulate the Nrf2/ARE pathway and repress NF-κB activation, reducing the expression of pro-inflammatory cytokines and COX-2, while strengthening enzymatic antioxidant defenses [124]. Recent advances in the broader pharmacological literature further expand the biological relevance of quercetin beyond its antioxidant and anti-inflammatory roles. A narrative review published in *Nutrients* (2025) highlights quercetin as a bioactive compound involved in women’s reproductive health, reporting modulatory effects on hormonal balance, fertility parameters, and gynecological disorders such as polycystic ovary syndrome, recurrent miscarriage, and endometrial cancer [125,126,127,128,129,130,131,132,133,134,135]. The authors describe molecular pathways through which quercetin may exert such effects, including PI3K/AKT, Nrf2, NF-κB, and other hormone-regulated signaling cascades, underscoring its ability to influence oxidative stress, inflammation, and endocrine regulation in reproductive tissues. These findings reinforce the broader pharmacological potential of quercetin as a principal phenolic constituent of *P. quinquefolia*, suggesting that its relevance may extend into reproductive and metabolic domains, although these hypotheses remain speculative until directly validated in this species. On the other hand, catechin and epicatechin, present in various Vitaceae species, exert antioxidant effects through direct redox activity and metal chelation and attenuate inflammation associated with oxidative stress and lipotoxic processes in cell and animal models [125]. In addition, kaempferol, identified in *Parthenocissus* species (specifically *P. tricuspidata*) [126], and widely distributed within the Vitaceae family, exhibits radical scavenging activity and modulates inflammatory pathways by inhibiting NF-κB and reducing mediators such as iNOS and COX-2 [127]. Taken together, this evidence suggests that the antioxidant and anti-inflammatory effects observed in *P. quinquefolia* may derive from the combined action of these flavonols, in interaction with catechins, on convergent molecular networks related to oxidative stress and inflammatory signaling. However, the specific participation of Nrf2 and the possible synergies between these compounds need to be evaluated directly in this species.

Beyond the polyphenolic profile of the photosynthetic organs, the biology of the tendrils and adhesion disks provides a distinctive chemical and structural component whose phytochemistry remains practically unexplored. Tendrils, described in Vitaceae for more than three centuries [128], are flexible, branched structures that, in species such as *P. quinquefolia*, develop expanded adhesive disks at the apexes, allowing attachment to the substrate by flattening and secreting mucilage [129,130]. In this species, adhesion does not depend on the coil, but on an adhesive composed of branched rhamnogalacturonan I, callose and mucilaginous pectins [131]. The formation of the disks involves the accumulation of mucilage, adjustment to the microrelief of the support, and subsequent lignification, which confers high environmental resistance [132]. Although tendrils are recognized as structures with medicinal value in several Vitaceae [133], to the best of our knowledge, there are no studies evaluating the bioactive properties of adhesive disks. This knowledge gap points to a concrete opportunity to investigate the relationship between its specialized polysaccharide composition and potential anti-inflammatory or antioxidant applications in *Parthenocissus*. Considering the range of antioxidant, anti-inflammatory and, in some cases, neuroprotective activities described for the flavonoids and stilbenoids present in *P. quinquefolia*, as well as the structural and polysaccharide properties of its tendrils and adhesive disks, the species emerges as a promising candidate for the development of standardized extracts, nutraceutical supplements and possible drugs derived from natural products. The combination of phenolic compounds, with extensive mechanistic support, and less-studied oligomeric metabolites suggests a synergistic potential that could be exploited in both anti-inflammatory interventions and strategies aimed at controlling oxidative stress or protecting tissues. However, moving towards biomedical applications requires validating the safety, bioavailability and in vivo efficacy of these compounds and their mixtures, as well as deepening the characterization of metabolites typical of specialized structures such as adhesion disks. The interpretation of the present findings requires consideration of biogeographical factors. All included studies were conducted on specimens collected in the Northern Hemisphere, where climatic conditions, photoperiod, temperature gradients and soil composition differ substantially from those in Chile. It is well-established that secondary metabolite biosynthesis in plants is modulated by environmental parameters such as UV exposure, temperature, water availability, altitude and soil nutrients, which may influence both the qualitative and quantitative profiles of phenolic compounds. Therefore, it is plausible that specimens of *P. quinquefolia* growing in Chile could exhibit distinct phenolic fingerprints either in relative abundance or in the presence of specific metabolites when compared to those reported in the literature. Comparative metabolomic studies would be required to confirm whether the stilbenoid and flavonoid profiles described in Northern Hemisphere populations are conserved under Chilean ecological conditions. In the Chilean context, a Mediterranean hotspot highly vulnerable to climate change and environmental oxidative stress, the characterization of antioxidant compounds from *P. quinquefolia* gains additional relevance [134,135]. Moreover, the widespread use of this species in nature-based solutions, especially in Mediterranean-type climates such as Chile, underscores the importance of understanding its phytochemical composition to more accurately assess its biological value and potential functional applications.

### 4.2. Limitations

Several methodological limitations should be considered when interpreting the findings of this review. First, the primary studies exhibited substantial heterogeneity in extraction procedures (e.g., solvent polarity, extraction temperature, ultrasound-assisted extraction), plant material selection (leaves, stems, fruits, roots), and phytochemical processing, all of which influence metabolite yield and composition. Second, analytical approaches varied across studies, ranging from qualitative phytochemical screening to quantitative chromatographic techniques (e.g., HPLC, HPLC-MS, GC-MS), limiting comparability of reported metabolite profiles. Third, biological assays used to assess antioxidant and anti-inflammatory activity were not standardized, with studies employing diverse in vitro systems (e.g., DPPH, ABTS, FRAP, NO scavenging, COX-2/iNOS expression assays), preventing direct aggregation of results. Fourth, the absence of in vivo or clinical studies restricts the translational interpretation of the reported bioactivities. Finally, the review design itself was limited by the lack of meta-analytic synthesis due to insufficiently homogeneous data and by the possibility of publication bias toward positive findings. Taken together, these factors warrant cautious interpretation and highlight the need for standardized, mechanistic, and in vivo investigations.

## 5. Conclusions

In conclusion, available evidence suggests that *P. quinquefolia* is a notable source of secondary metabolites (particularly polyphenols, flavonoids, and stilbenoids) with documented chemoprotective, antioxidant, and anti-inflammatory activities. These properties underscore its value not only as an ornamental or ethnobotanical species but also as a promising model for contemporary biomedical research. The polyphenols characterized in the species, including catechin, epicatechin, quercetin, myricetin, resveratrol and several resveratrol-derived oligomers (parthenocissins), act on central molecular pathways such as NF-κB, NLRP3, MAPKs, eNOS and Nrf2, thereby modulating oxidative stress and chronic inflammatory responses; processes implicated in cardiovascular, metabolic and neurodegenerative diseases, as well as in oncogenesis and aging. Although the existing body of literature (14 studies) is primarily based on in vitro assays and still lacks validation in animal models or clinical settings, the findings collectively suggest that *P. quinquefolia* is a credible natural source for the development of extracts, nutraceutical formulations, and lead compounds for drug discovery. Advancing towards translational applications will require rigorous studies addressing bioavailability, safety and in vivo efficacy to substantiate its therapeutic potential in conditions driven by oxidative stress and inflammation.

## Figures and Tables

**Table 1:** General characteristics of each selected study (n = 14).

Study	Plant Part Used	Extraction Method	Analytical Technique	Identified Compounds	Concentrations	Assay Conditions/Biological Assays
Tanaka T., Inuma M. Murata H. (1998) [23]	Stems	Acetone + Methanol extracts	Phytochemistry/Chromatography	Parthenocissin A, Parthenocissin B	Not reported	Antioxidant activity (in vitro), qualitative
Kumar S. et al. (2011) [9]	Not specified	Not reported	Not reported	Not reported	250 mg/kg p.c. (oral)	Antidiabetic activity in Zucker rats
Yang J. et al. (2013) [24]	Stems	Ethanol extract	HPLC/Chromatography	Parthenocissin M, Parthenocissin N, Miyabenol C, ε-viniferin	Not reported	Hepatoprotective, antibacterial, antifungal assays (in vitro)
Rattanata N. et al. (2014) [25]	Leaves	Ethanol extract	Colorimetric assays	Total phenolics, flavonoids (non-specific)	TPC/FC values not stated in manuscript	Antioxidant (DPPH, ABTS), Antibacterial assays
Zardi-Bergaoui A. et al. (2016) [6]	Pods & seeds	Soxhlet extraction (hexane)	GC-MS	Linoleic acid, Palmitic acid, Oleic acid	% composition reported in original study (not provided in manuscript)	Antioxidant/Antiradical activity
Deshmukh-Omraj S. (2017) [26]	Roots	Crude extract	Qualitative phytochemical screening	Alkaloids, Flavonoids, Terpenoids, Steroids, Coumarins, Carbohydrates, Tannins	Not applicable (qualitative)	Antibacterial assays (in vitro)
Zaheer-Ud-Din K. et al. (2017) [7]	Leaves & fruits	Crude ethanolic extracts	Qualitative phytochemical screening	Terpenoids, Flavonoids, Saponins, Tannins, Alkaloids, Glycosides	Not applicable (qualitative)	Antioxidant assays (DPPH)
Faisal S. et al. (2018) [10]	Bark	Methanolic extract	Colorimetric assays	Total phenolics	TPC value not provided in manuscript	Free radical scavenging assays
Mohamed A. A. et al. (2021) [27]	Fruits	Ethanolic extract	HPLC	Rutin, Myricetin	Rutin: 1891.60 mg/100 g extract; Myricetin: 241.06 mg/100 g extract	Antimicrobial and fungicidal assays
Ticha et al. (2017) [28]	Fruits	Ultrasonic aqueous extraction	HPLC-MS	Anthocyanins: delphinidin, petunidin, cyanidin, malvidin, peonidin, pelargonidin	Not reported	No biological assays performed (phytodyeing application)
Makhynia L., Yemelianova O. (2023) [29]	Leaves (flowering & fruiting stages)	Not reported	Qualitative phytochemical screening	Phenols, Flavonoids, Polysaccharides, Saponins, Tannins, Catechins, Anthocyanins, Hydroxycinnamic acids	Not applicable	No biological assays performed
Konovalova O. et al. (2023) [30]	Leaves, shoots, fruits	Ethanol extract	HPLC	Rutin, Quercetin, Quercetin-3-β-glycoside, Naringin (leaves & shoots); Epicatechin, Catechin, Gallocatechin, Epicatechin gallate, Luteolin (fruit)	Not reported	Antioxidant assays (DPPH)
Rónavári A. et al. (2023) [31]	Leaves	Green extract for nanoparticle synthesis	HPLC (phenolics), LC-analysis	Total phenolics, sugars (fructose, glucose, sucrose, mannitol, citric acid)	Not reported	No biological assays performed
Önder FC. et al. (2024) [11]	Fruits & red leaves	Ethanol extract	HPLC	Total phenolic content (non-specific)	TPC reported in original study (not in manuscript)	Anticancer, Antioxidant, Antimicrobial assays

**Table 2:** Phytochemical and bioactivity characteristics specific to each selected study.

Study	Plant Part	Identified Compounds	Quantification	Bioassay Type	Biological Effect
Tanaka T. Inuma M., Murata H. (1998) [23]	Stems	Parthenocissins A, B	Not reported (structural identification only)	Antioxidant (in vitro)	Positive antioxidant activity
Kumar S. et al. (2011) [9]	Not specified	Not reported	Not reported	In vivo (Zucker diabetic rat model; Oral glucose tolerance; Insulin ELISA)	Positive antihyperglycemic effect
Yang J. et al. (2013) [24]	Stems	Parthenocissins M/N, Miyabenol C, ε-viniferin	Not reported	Chemopreventive, antibacterial, antifungal	Positive effects reported
Rattanata N. et al. (2014) [25]	Leaves	Total phenolics/flavonoids (nonspecific)	TPC/TFC not reported	Antioxidant (DPPH, ABTS), antibacterial	Positive activity
Zardi-Bergaoui A. et al. (2016) [6]	Pods and seeds	Linoleic, palmitic, oleic acids	inhibition DPPH 31.6–83.8%	Antioxidant/antiradical	Positive activity
Deshmukh-Omraj S. (2017) [26]	Roots	Various phytochemicals (qualitative)	Not applicable (qualitative)	Antibacterial	Positive activity
Ud-Din Khan Z. et al. (2017) [7]	Leaves & fruits	Terpenoids, flavonoids, etc. (qualitative)	Not applicable (qualitative)	Antioxidant (DPPH)	Positive activity
Faisal S. et al. (2018) [10]	Bark	Total phenolics	IC_50_ bark ~24.32 mg/mL; stem ~13.6 mg/mL	Antioxidant	Positive scavenging activity
Mohamed A. A. et al. (2021) [27]	Fruits	Rutin, Myricetin	Not reported	Antimicrobial/fungicidal	Positive activity
Ticha et al. (2017) [28]	Fruits	Anthocyanins (delphinidin, petunidin, cyanidin, etc.)	Not reported	Not evaluated	No bioactivity reported
Makhynia L., Yemelianova O. (2023) [29]	Leaves (2 stages)	Phenolics, flavonoids, etc. (qualitative)	Not applicable (qualitative)	Not evaluated	No bioactivity reported
Konovalova O. et al. (2023) [30]	Leaves/shoots/fruits	Rutin, quercetin, catechins, etc.	Not reported	Antioxidant (DPPH)	Positive activity
Rónavári A. et al. (2023) [31]	Leaves	Total phenolics & sugars	Not reported	Not evaluated	No bioactivity reported
Cömert Önder F. et al. (2024) [11]	Fruits & red leaves	Total phenolics	TPC reported in original study (Not in manuscript)	Anticancer, antioxidant, antimicrobial	Positive activity

## Data Availability

No new data were created or analyzed in this study. Data sharing does not apply to this article.

## Supplementary Materials

The following supporting information can be downloaded at https://www.mdpi.com/article/10.3390/antiox15020169/s1.

## Abbreviations

PRISMA	Preferred reporting items for systematic reviews and meta-analyses
OSF	Open science framework
PCs	Phenolic compounds
N	Nitrogen
S	Sulphur
ROS	Reactive oxygen species
NF-κB	Nuclear factor kappa B
NO	Nitric oxide
eNOS	Endothelial nitric oxide synthase
TGF-β	Transforming growth factor-beta
LPS	Lipopolysaccharide
TNFα	Tumor necrosis factor-alpha
CRP	C-reactive protein
SAA	Serum amyloid A
ORAC	Oxygen radical absorbance capacity
AP-1	Activator protein 1
MCP-1	Monocyte chemotactic protein 1
MMP-2	Matrix metalloproteinase-2
NLRP3	NOD-like receptor heat domain-associated protein 3
IL	Interleukin
ATP	Adenosine triphosphate
iNOS	Inducible nitric oxide synthase
COX	Cyclooxygenase
RNS	Reactive nitrogen species
SOD	Superoxide dismutase
CAT	Catalase
Nrf2	Nuclear factor erythroid 2-related factor 2
HIV	Human immunodeficiency virus
HBV	Hepatitis B virus
HSV	Herpes simplex virus
LDL	Low-density lipoprotein
NASH	Non-alcoholic steatohepatitis
MAPKs	Mitogen-activated protein kinas
RCS	Reactive chlorine species
HO-1	Heme oxygenase-1
PPARγ	Peroxisome proliferator-activated receptor gamma
SIRT1	Sirtuin-1
PGE2	Prostaglandin E2
CLA	Conjugated linoleic acid
ARE	Antioxidant response element
