# Supplementary material for: Bioactive Components of Parthenocissus quinquefolia with Antioxidant and Anti-Inflammatory Properties: A Systematic Review
Source: Antioxidants (Basel). 2026 Jan 27;15(2):169. doi: 10.3390/antiox15020169 (PMC12937967; doi:10.3390/antiox15020169)
Supplement: Supplementary file 1 [file antioxidants-15-00169-s001.zip › antioxidants-4101868-supplementary.pdf]

Supplementary Table S1: Details of the search strategy

Database	Search strategy	Results	
		20-10-25	28-12-2025
**Medline**	Search: ((((((Nrf2/ARE pathway[Title/Abstract]) OR (NF-κB signaling[Title/Abstract])) OR (secondary metabolites[Title/Abstract])) AND (phenolic profile[Title/Abstract])) OR (phyto-chemical analysis[Title/Abstract]))<br><br>(("nrf2 are pathway"[Title/Abstract] OR "nf kappab signaling"[Title/Abstract] OR "secondary metabolites"[Title/Abstract]) AND "phenolic profile"[Title/Abstract]) OR "phyto chemical analysis"[Title/Abstract]	57	57
**Wos**	Nrf2/ARE pathway OR NF-κB signaling OR secondary metabolites AND phenolic profile OR phyto-chemical analysis	41	41
**CINAHL**	Nrf2/ARE pathway OR NF-κB signaling OR secondary metabolites AND phenolic profile OR phyto-chemical analysis	40	40
**SCOPUS**	Nrf2/ARE pathway OR NF-κB signaling OR secondary metabolites AND phenolic profile OR phyto-chemical analysis	165	167
**Google Scholar**	Nrf2/ARE pathway OR NF-κB signaling OR secondary metabolites AND phenolic profile OR phyto-chemical analysis	360	362
	Total	663	665

*All searches were conducted on december 28, 2025.
